# Alterations in white matter network dynamics in patients with schizophrenia and bipolar disorder

**DOI:** 10.1002/hbm.25892

**Published:** 2022-05-13

**Authors:** Bin Wang, Shanshan Zhang, Xuexue Yu, Yan Niu, Jinliang Niu, Dandan Li, Shan Zhang, Jie Xiang, Ting Yan, Jiajia Yang, Jinglong Wu, Miaomiao Liu

**Affiliations:** ^1^ Department of Information and Computer Taiyuan University of Technology Taiyuan China; ^2^ Department of Medical Imaging The Second Hospital of Shanxi Medical University Taiyuan China; ^3^ Teranslational Medicine Research Center Shanxi Medical University Taiyuan China; ^4^ Graduate School of Interdisciplinary Science and Engineering in Health Systems Okayama Japan; ^5^ School of Psychology Shenzhen University Shenzhen China

**Keywords:** classification, control strategy, controllability, supercontroller, synchronizability, tradeoff

## Abstract

Emerging evidence suggests white matter network abnormalities in patients with schizophrenia (SZ) and bipolar disorder (BD), but the alterations in dynamics of the white matter network in patients with SZ and BD are largely unknown. The white matter network of patients with SZ (*n* = 45) and BD (*n* = 47) and that of healthy controls (HC, *n* = 105) were constructed. We used dynamics network control theory to quantify the dynamics metrics of the network, including controllability and synchronizability, to measure the ability to transfer between different states. Experiments show that the patients with SZ and BD showed decreasing modal controllability and synchronizability and increasing average controllability. The correlations between the average controllability and synchronizability of patients were broken, especially for those with SZ. The patients also showed alterations in brain regions with supercontroller roles and their distribution in the cognitive system. Finally, we were able to accurately discriminate and predict patients with SZ and BD. Our findings provide novel dynamic metrics evidence that patients with SZ and BD are characterized by a selective disruption of brain network controllability, potentially leading to reduced brain state transfer capacity, and offer new guidance for the clinical diagnosis of mental illness.

## Graphical abstract

We used dynamics network control theory to quantify the dynamics metrics of the network, including controllability and synchronizability of healthy controls, patients with schizophrenia and bipolar disorder. Our findings provide novel dynamic metrics evidence that patients with schizophrenia and bipolar disorder are characterized by a selective disruption of brain network controllability, potentially leading to reduced brain state transfer capacity, and offer new guidance for the clinical diagnosis of mental illness.

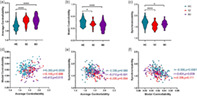

## 1 INTRODUCTION

The brain's white matter tracts form a large‐scale wiring diagram or network that has an impact on cognitive functions (Filley & Fields, 2016), development (Imperati et al., 2011), disease (Lenka et al., 2020), and rehabilitation (Izadi‐Najafabadi & Zwicker, 2021). Despite the intuitive relationship between network topology and brain function (Sporns, 2013), understanding the complex patterns of white matter network dynamics could inform cognitive dysfunction that accompany altered wiring patterns (Jeganathan et al., 2018; Lee et al., 2019; Medaglia, 2019).

Schizophrenia (SZ) and bipolar disorder (BD) are characterized by symptoms in a range of behavioral, cognitive, and affective dysfunction (Baker et al., 2019; Keshavan et al., 2011; Schretlen et al., 2007), and may relate to disconnectivity among brain regions (Cui et al., 2011; Klauser et al., 2017; Schneiderman et al., 2011; Skudlarski et al., 2013; Yan et al., 2018). Emerging evidence suggests that the brain's wiring organization is disrupted in SZ (for review see Bassett et al., 2008; Kelly et al., 2018) and BD (Roberts et al., 2018; Wang et al., 2019). Braun suggest these mental disease may involve anatomical network dynamics disruption, involving the control ability of different dynamic state transitions (Braun et al., 2021). The network dynamics disruption might be the primary cause of the disease (Braun et al., 2021; Zöller et al., 2021).

The research of network dynamics is one of the frontiers in neuroscience. Recent studies have demonstrated that the structural connection determines network dynamics configuration of human brain (Betzel et al., 2016; Patankar et al., 2020). Network dynamics can be used to study how the connection mode between elements restricts the complex physiological activities of the nervous system. The network dynamics characteristics can be captured by two inverted metrics: controllability (Gu et al., 2015) and synchronizability (Tang et al., 2017). These two concepts can be used to examine how brains might be optimized for different types of dynamics. Controllability is a structural predictor that predicts the ability to switch from one dynamic state to another (Gu et al., 2015). The average controllability and modal controllability, respectively, describe the ability to drive brain states to easy‐to‐reach or difficult‐to‐reach states, where the ease or difficulty is determined by the amount of energy input. Synchronizability describes the ability of regions in the network to support the same temporal dynamical pattern (Tang et al., 2017). These dynamic measures of the white matter network have provided a robust and biologically plausible mechanism for determining how damage to the brain structural organization may diminish brain cognition (Kang et al., 2019; Lee et al., 2019). Network controllability measurement can provide characteristic attributes for different cognitive brain systems (Gu et al., 2015). These characteristics change with development (Cui et al., 2020; Tang et al., 2017). They are reliable and heritable attributes of structural connectors (Lee et al., 2019), and can track individual characteristics in cognitive function (Lee et al., 2019), executive function (Cui et al., 2020) and impulse (Cornblath et al., 2019). Moderate synchronizability has been shown to be conducive to the transformation of neural activity (Tang et al., 2017; Vuksanović & Hövel, 2015). Zhu et.al found SZ involves a disruption of neural synchronizability from the perspective of network properties (Zhu et al., 2020) Controllability characteristic research methods have proved particularly useful in detecting brain function in health and disease (Bernhardt et al., 2019; Tang et al., 2017). However, whether dynamic abnormalities of the white matter network are present in SZ and BD patients remains to be determined.

Here, we capitalize on recent theoretical advances in dynamic network control theory to investigate alterations in changes and constrain patterns of dynamics in the brain of patients with SZ and BD. Using neuroimaging data in the SZ, BD, and normal control groups, we examined how white matter network abnormalities lead to abnormal dynamic network characteristics in patients. The average controllability, modal controllability, and synchronizability of the white matter network were analyzed at the global and regional levels. In addition, the relationship between controllability and synchronizability and dysfunction in clinical symptoms were further studied.

## 2 MATERIALS AND METHODS

### 2.1 Participants

All imaging and psychiatric symptom data were acquired from the UCLA Consortium for Neuropsychiatric Phenomics LA5c Study, and the data set is hosted on OpenfMRI (www.openfmri.org) (Poldrack et al., 2016) under the accession number ds000030. The current study included 105 healthy controls (male/female: 58/47, 21–49 years), 45 patients with SZ (male/female: 34/11, 22–49 years), and 47 patients with BD (male/female: 28/19, 21–50 years).

### 2.2 Imaging data preprocessing

MRI data were acquired on one of 3 T Siemens Trio scanners, located at UCLA. Structural MRI data were acquired on one of two 3 T Siemens Trio scanners located at UCLA. Diffusion‐weighted imaging (DWI) data were obtained using echo‐plane sequence, and the parameters were as follows: 64 directions, 2 mm slices, TR/TE = 9000/93 ms, 1 average, 90 degree flip angle, 96 × 96 matrix, axial slices, and *b* = 1000 s/mm^2^. In addition to diffusion scans, T2‐weighted sagittal sequence images of the whole brain were obtained by a magnetization‐prepared rapid acquisition gradient‐echo sequence, and the parameters were as follows: TR = 1.9 s, TE = 2.26 ms, matrix = 256 × 256, FOV = 250 mm, sagittal plane, slice thickness = 1 mm, and 176 slices.

The image preprocessing steps were performed using the PANDA toolbox and FSL 5.0 (https://fsl.fmrib.ox.ac.uk/fsl/). First, the fractional anisotropy (FA) was calculated for each voxel. The FA images in the native space were coregistered to T1‐weighted images by an affine transformation. Then, structural images were nonlinearly registered to the ICBM152 template. An inverse warping transformation from the standard space to the native MRI space was obtained. Based on this inverse transformation, the BN atlas in the standard space could be inversely warped back to the individual native space. In the end, the deterministic fiber tracking algorithm is used to reconstruct the fiber path. The fiber tracking procedure started from the deep WM regions and terminated when the intersection Angle of two continuous moving directions was >35° or the FA exceeded the threshold range (0.1–1) (Cui et al., 2013). The number of streamlines between two regions was defined as the network edge based on the human Brainnetome Atlas (BN) parcellation scheme (Fan et al., 2016).

### 2.3 Connectome construction

In this study, network construction was based on deterministic fiber tracking of the white matter network. The Structure connection matrix for each subject was constructed using the PANDA in MATLAB R2015b. First, the whole brain was parcellated into 246 regions (nodes) based on the human Brainnetome Atlas (BN) parcellation scheme (Fan et al., 2016), which contains cortical and subcortical regions. A linear transformation was applied locally within each subject's DTI image correlated with the T1‐weighted image to coregister them to the b0 image with DTI space, and then a nonlinear transformation was applied to map to the ICBM152 T1 template (Montreal Neurological Institute [MNI]). Then, the subject‐specific BN mask was weaved from the MNI space to the DTI native space with the corresponding inverse transformation. Each brain region was defined as one node. The edge weight $A_{\mathrm{ij}}$ in the adjacency matrix A were defined by the number of streamlines connecting each pair of nodes end‐to‐end.

Brain regions within the 246‐region parcellation were mapped to seven functional networks defined by Yeo et al.(Yeo et al., 2011):visual, somatomotor, dorsal attention, ventral attention, limbic, frontal–parietal and default mode.

### 2.4 Dynamics model

To better understand the dynamic characteristics of neural networks, we combine network control theory with brain dynamics and use a linear dynamic model to simulate the nonlinear dynamic process of brain neural activities. Previous studies have proved that this model can predict the differences in neural network dynamics (Gu et al., 2015).

Then, we introduce a simplified noise‐free linear discrete‐time and time‐invariant network model:

(1) $$x\left(t+1\right)=\mathrm{Ax}\left(t\right)+B_{K}u_{K}\left(t\right),$$

where *x*: $R_{\geq 0}\to R^{N}$ describes the state of brain regions over time, and each state is the intensity of neurophysiological activity across brain regions at a single time point. $A\in R^{N\times N}$ is the symmetric adjacency matrix, in which elements $a_{\mathrm{ij}}$ indicate the number of white matter streamlines connecting two different brain regions. The size of vector x is determined by the number of brain regions divided. In my study, this template is 246 partitions, and the value of the vector x is the intensity of activity across brain regions of the BOLD signal. The values of the diagonal elements of matrix A are all 0, that is $A_{\mathrm{ii}}=0$. Note that to ensure stability, we divide the matrix by $\;1+\delta_{0}\left(A\right)$, where $\delta_{0}\left(A\right)$ is the largest singular value of A. The input matrix $B_{K}$ identifies the control points *K* in the brain, where *K* = {*k*_1_, …, *k*_*m*_}:

(2) $$B_{K}=\left[e_{k_{1}},\ldots ,e_{k_{m}}\right],$$

and $e_{i}$ denotes the *i*th canonical vector of dimension N. The input $u_{K}$: $R_{\geq 0}\to R^{m}$ denotes the control strategy. We use the invertibility of the Gramia matrix $W_{K}$ to guarantee the controllability of the results of Equation (1) (Basile, 1971), where:

(3) $$W_{K}=\sum_{\tau =0}^{\infty }A^{\tau }B_{K}B_{K}^{T}A^{\tau }.$$

We control one node at a time through the matrix B.

### 2.5 Network controllability and synchronizability metrics

In terms of network controllability, we study two control diagnoses: controllability and synchronizability, that describe the magnitude of the ability to drive brain states to different states in a particular fashion (Gu et al., 2015). Average controllability refers to the ability of a brain region to transfer brain states to easily reachable states. In other words, average controllability of a network equals the average input energy applied to a set of control nodes required to reach all possible target states. Average input energy is proportional to Trace $\left(W_{K}^{-1}\right)$, the trace of the inverse of the controllability Gramian, and we adopt Trace ($W_{K}$) as a measure of average controllability (Gu et al., 2015), due to the Trace $\left(W_{K}^{-1}\right)$ tends to be very ill‐conditioned. And modal controllability refers to the ability of a brain region to transfer brain states to difficult‐to‐reach states. Modal controllability refers to the ability of nodes to control the transformation of dynamic network to various evolution modes (Hamdan & Nayfeh, 1989), and modal controllability is computed from the eigenvector matrix $V=\left[v_{\mathrm{ij}}\right]$ of the network adjacency matrix A. If $v_{\mathrm{ij}}$ is small, then the *j*‐th evolutionary mode of the input‐independent form is poorly controllable from node *i*. Modal controllability was defined as $\emptyset_{i}=\sum_{j=1}^{N}\;\left(1-\lambda_{j}^{2}\;\left(A\right)\right)v_{\mathrm{ij}}^{2}$ as a scaled measure of all N modes $\lambda_{1}\left(A\right),\ldots \lambda_{N}\;\left(A\right)$ of the brain region i (Pasqualetti et al., 2014).

Synchronizability measures the ability of a network to persist in a single synchronous state. Following Tang et al., (2017), linear stability depends on the positive eigenvalues {λ_*i*_}, *i* = 1, …, *N* − 1, of the Laplacian matrix L defined by $L_{\mathrm{ij}}=\delta_{\mathrm{ij}}\sum_{k}A_{\mathrm{ik}}-A_{\mathrm{ij}}$. The condition for stability depends on whether these eigenvalues fall into the stable region and the shape of MSF. Hence, we can use the normalized spread of the eigenvalues to quantify how synchronizable the network will generally be (Nishikawa & Motter, 2010; Tang et al., 2017). We therefore quantify network synchronizability as:

(4) $$\frac{1}{\sigma^{2}}=\frac{d^{2}\left(N-1\right)}{\sum_{i=1}^{N-1}{\left|\lambda_{i}-\bar{\lambda }\right|}^{2}},\;\text{where}\;\bar{\lambda }=\frac{1}{N-1}\sum_{i=1}^{N-1}\lambda_{i},$$

and $d=\frac{1}{N}\sum_{i}\sum_{j\neq i}A_{\mathrm{ij}}$, the average coupling strength per node, which normalizes for the overall network strength. We calculate the synchronizability of each region according to the split of Equation (4).

### 2.6 Experimental design

This experiment tested the dynamics indexes of three experimental groups: controllability and synchronizability. *T*‐test was used to compare the significance of differences between the SZ and HC groups, BD and HC groups, SZ and BD groups in these three indicators. The difference of the correlation between the three groups was analyzed by linear fitting. After selecting the three brain regions with higher indexes as super control brain regions, the differences of super control brain regions in the three experimental groups were compared. The *t*‐test was used to compare the brain regions with significant differences in controllability and synchronizability among the three groups, calculate their Spearman correlation with clinical symptoms, and control age and sex. Finally, the ability to classify patients and healthy people and predict clinical scores are analyzed through the indicators of controllability and synchronizability.

### 2.7 Classification of participants

In the final analyses, we used multivariate metrics to identify the distinctions between the HC and SZ, HC and BD, and SZ and BD groups, enabling subject‐specific group assignment based on three types of data as features: average controllability, modal controllability and synchronizability. We employed linear kernel‐based support vector machine of LIBSVM software library for classification, and the parameter boundary is (0,1). (https://www.csie.ntu.edu.tw/~cjlin/libsvm/). Then, the support vector machine is trained by knowing the value of the classification results.

Before classification, we extracted individual data and data with significant differences between group features to improve performance. We applied k cross‐validations, iteratively dividing the data into separate training and testing sets. Finally, ROC curve is used to evaluate the performance of the classifier.

### 2.8 Prediction model based on dynamic metrics

To determine whether the dynamic metrics can predict the scale scores, we used the improved connectome‐based predictive modeling (CPM) method. In this model, we used leave‐one‐subject‐out cross‐validation, and the model was trained on the controllability and synchronizability values and scale scores of *n* − 1 participants and tested on the left‐out participant. This MATLAB script is available from https://www.nitrc.org/projects/bioimagesuite/ (Shen et al., 2017).

We briefly describe the process of the improved CPM method here. In the first step, in each person's node‐controllability matrix and node‐synchronizability matrix (M*N), *M* is the number of nodes, and *N* is the number of subjects. The scale vector (N*1) was associated with the controllability value and synchronizability value of each node by a Spearman correlation in the above matrix. Next, only nodes that were significantly positively and negatively correlated with scale were retained (*p* < .05), and then the new matrix was normalized by the Z‐score. Then, the multiple linear regression model was used to estimate the relationship between the score of the prediction scale and the real scale by combining the positive and negative correlation characteristics. Finally, the predictive power of the model was measured by the Spearman correlation coefficient between the predicted scale score and the real scale score. All statistical tests were two‐tailed.

### 2.9 Statistical analysis

All statistical analyses were performed using the Statistical Package for Social Science (SPSS, v19.0). All the metrics are calculated by MATLAB. We conducted ANOVA tests to compare patients with SZ, those with BD, and healthy controls (HC) on the dynamic metrics: average controllability, modal controllability and synchronizability. We calculated the correlation between the three metrics and analyzed the correlation of metrics. To study the significant differences between groups, a two‐tailed t‐test was conducted to control for age and sex. The problem of multiple comparisons at the nodal level was addressed using Bonferroni correction method (Bonferroni *p* < .05). The Spearman correlation analysis was used to explore the relationship between the metrics and clinical symptoms in patients, with age and sex as covariates. The area under the curve (AUC) is used to evaluate the classification performance of vector machines.

## 3 RESULTS

### 3.1 The global dynamic metrics of white matter networks

**FIGURE 1:**
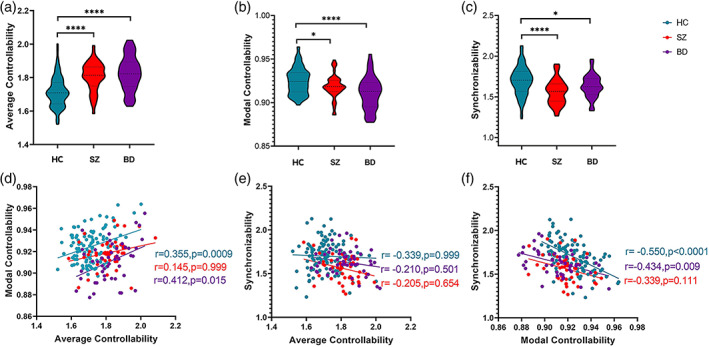
The average controllability, modal controllability and synchronizability and their correlation. (a, b, c) average controllability, modal controllability and synchronizability in HC, SZ and BD. The dotted lines represent the median and the quartile, and * indicates a significant difference between groups (*p < .05, ****p < .0001). (d, e, f) The correlation between controllability and synchronizability. The r values and p values are obtained by controlling for age and sex, and the *p* values are corrected by multiple testing. BD, bipolar disorder (purple); HC, healthy controls (blue); SZ, schizophrenia (orange).

As shown in Figure 1, significant group differences were observed in the average controllability, modal controllability and synchronizability (*F* = 11.821, *p* = .0001). Compared with the HC group, there was significantly higher average controllability in patients with SZ and BD (Figure 1a) (*t* = 5.828, *p* = .0001). The modal controllability (Figure 1b) and synchronizability (Figure 1c) were significantly lower in patients with SZ and BD (*t* = 2.459, *p* = .0151).

### 3.2 Relationship among the global dynamic metrics

Modal controllability was significantly positively correlated with average controllability (*r* = .412, *p* = .015) and negatively correlated with synchronizability (*r* = −0.434, *p* = .009) in the HC and BD groups and weaker in the BD group (Figure 1d,f). There was no significant correlation in the SZ group (*r* = .145, *p =* .111). No significant correlation between synchronizability and average controllability was found in the three groups (Figure 1e).

### 3.3 Comparison of the brain regions with supercontroller roles

**FIGURE 2:**
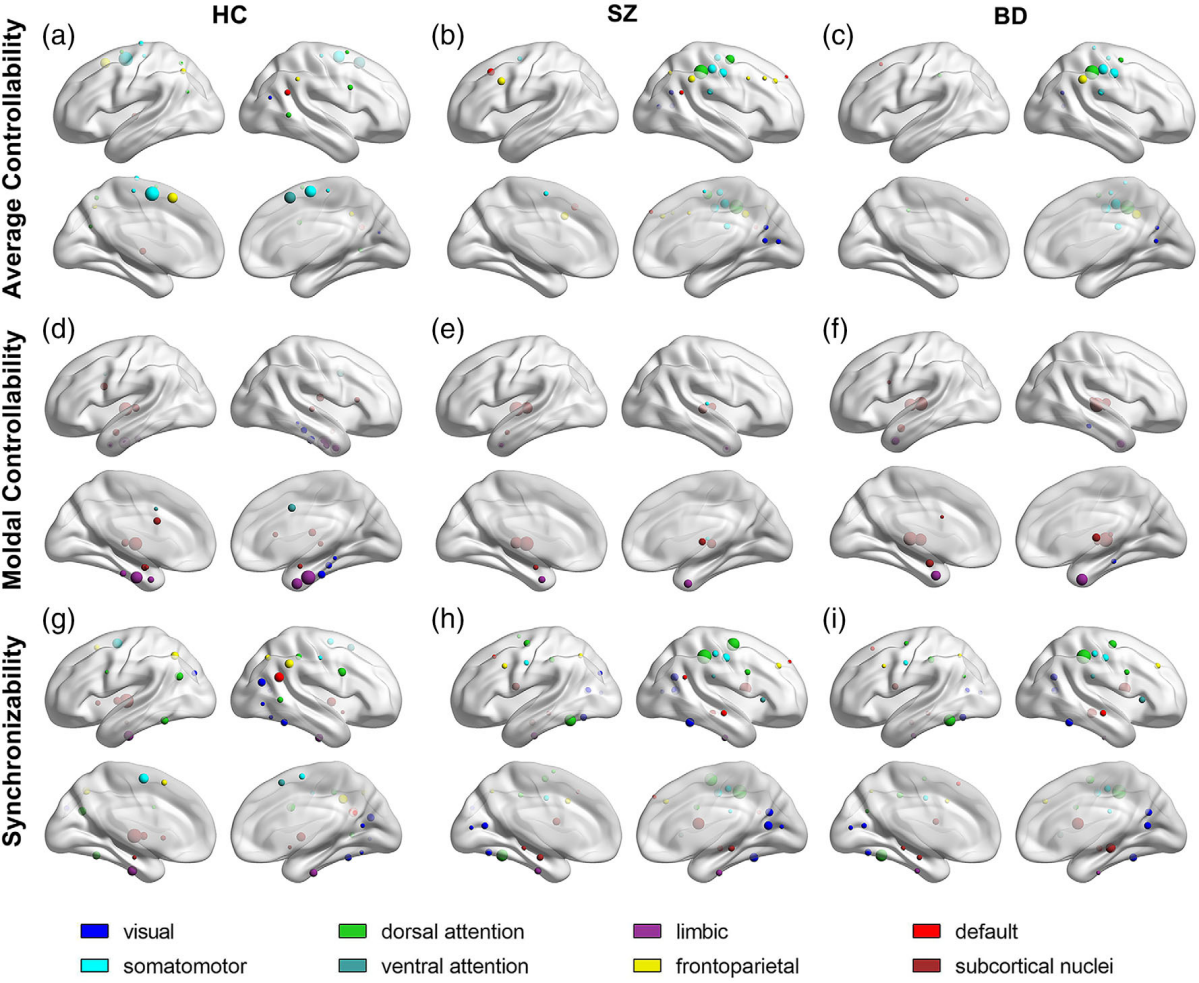
The values of the average controllability, modal controllability and synchronizability are higher than the mean plus the standard deviation in the HC, SZ, and BD groups. (a, b, c) Brain regions with high average controllability in the three groups; (d, e, f) brain regions with higher modal controllability in the three groups; and (g, h, i) brain regions with higher synchronizability in the three groups.

We determined the brain regions with supercontroller roles in the brain network (Figure 2), which also implies alteration of the control strategy for the cognitive system. The brain regions with the supercontroller regarding the average controllability varied greatly among patients with SZ and BD (Figure 2a,b,c) in the default, visual, limbic, frontal parietal, somatomotor networks. Compared with SZ, BD had more supercontroller regions in the somatomotor and dorsal attention systems but fewer supercontroller regions in the frontal parietal system. For modal controllability, the brain regions with the supercontroller role of patients with SZ and BD were distributed in the visual, ventral attention, and subcortical nuclei systems (Figure 2d,e,f). Compared with SZ, BD had more regions in the visual and subcortical nuclei system but fewer regions in the somatomotor systems. The brain regions with the supercontroller role in terms of synchronizability were widely distributed in all cognitive systems and altered slightly in patients with SZ and BD (Figure 2g,h,i).

### 3.4 Abnormal regional controllability and synchronizability in patients

**FIGURE 3:**
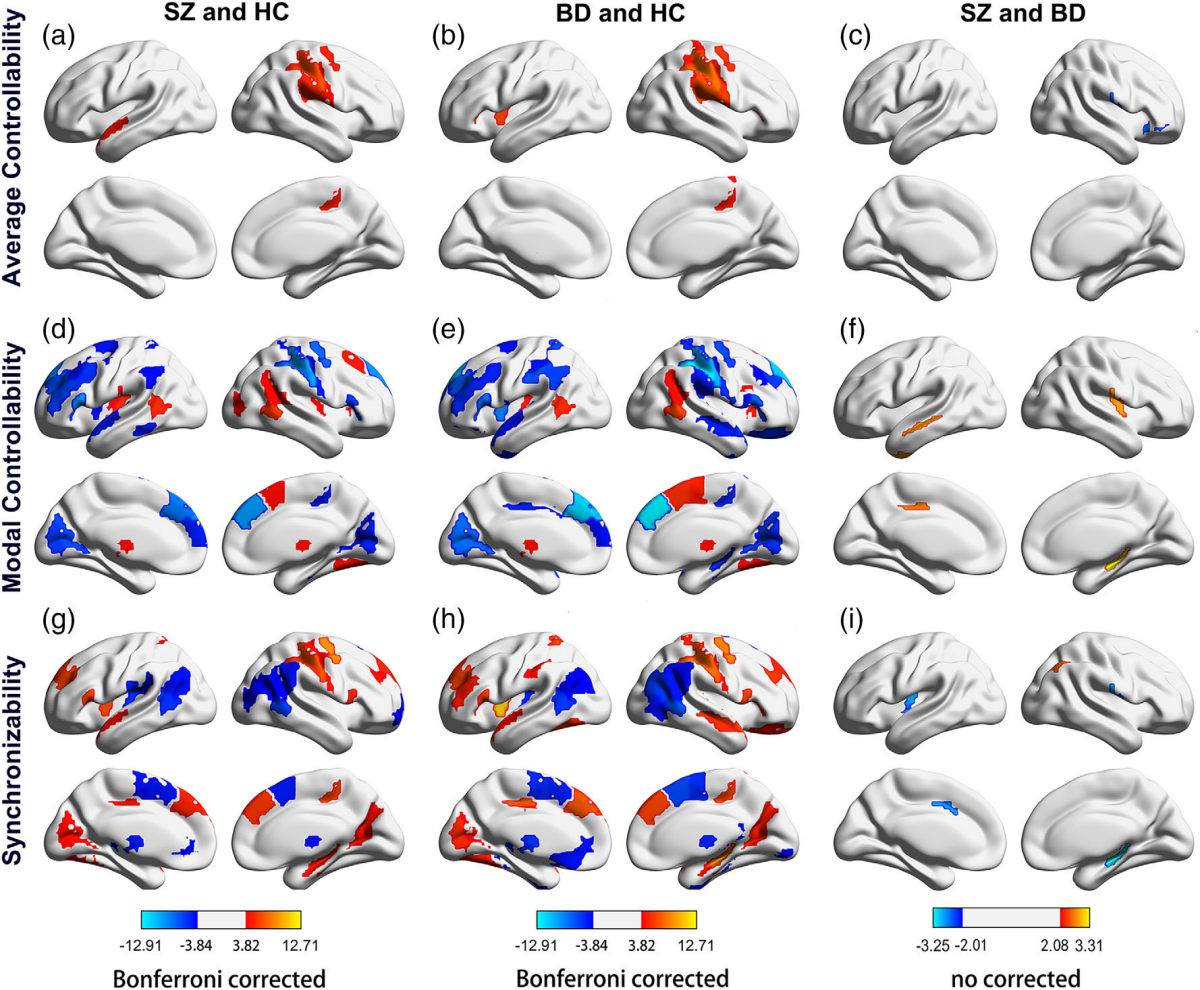
Brain regions with significant differences in controllability and synchronizability in patients with SZ and BD compared with the HC group. (a, b, c) Brain regions with significant differences in average controllability, modal controllability, and synchronizability between the SZ group and HC group; (d, e, f) brain regions with significant differences in average controllability, modal controllability and synchronizability between the BD group and HC group. *p* < .05 after the Bonferroni correction. (g, h, i) Brain regions with significant differences in average controllability, modal controllability and synchronizability between the SZ group and BD group. Warm colors indicate high controllability or synchronizability of patients with mental illness, while cool colors indicate low controllability or synchronizability of patients with mental disorders. BD, bipolar disorder; HC, healthy controls; SZ, schizophrenia.

**FIGURE 4:**
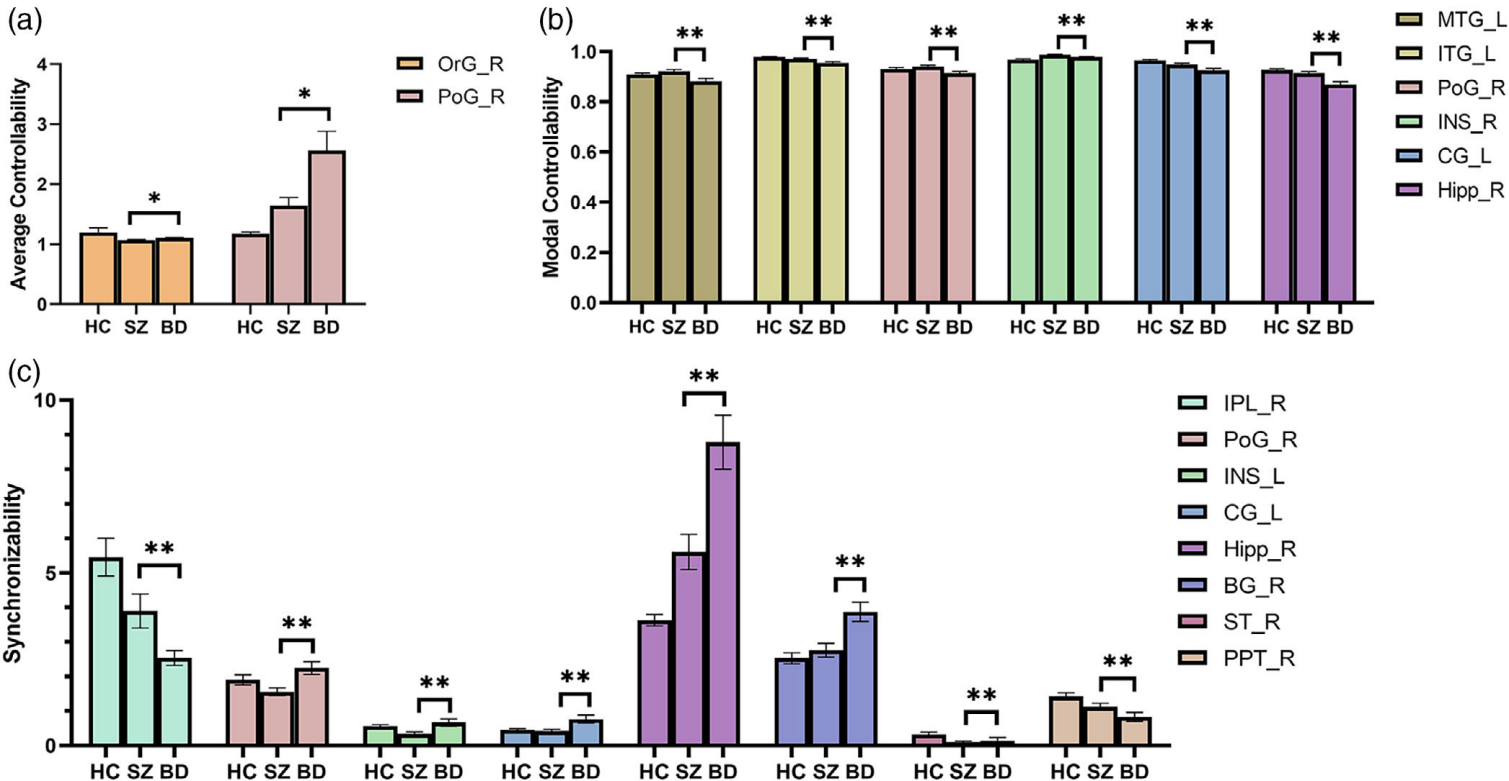
Brain region with differences in average controllability, modal controllability, and synchronizability between SZ and BD (no corrected). (a) Average controllability; (b) Modal controllability; and (c) Synchronizability. BG, basal ganglia; CG, cingulate gyrus; Hipp, hippocampus; INS, insular gyrus; IPL, inferior parietal lobule; ITG, inferior temporal gyrus; L, left; MTG, middle temporal gyrus; OrG, orbital gyrus; PoG, postcentral gyrus; PPT, posterior parietal thalamus; R, right; ST, sensory thalamus.

Given the global trends of increasing average controllability and decreasing modal controllability and synchronizability in patients, it is necessary to ask whether patients with mental illness are driven by specific regions of the brain, or whether all regions have different driving effects. Compared with the HC group, patients with SZ and BD showed significantly larger average controllability and synchronizability in the parietal lobe and significantly lower modal controllability (Figure 3). The regions with decreased modal controllability and increased synchronizability were broadly distributed in the frontal lobe, temporal lobe, parietal lobe, and occipital lobe. In most of the regions in the subcortical nucleus with significant differences, the modal controllability increased significantly, and the synchronizability decreased significantly. There was no significant difference after correction between patients with BD and those with SZ (Figure 3). Although we found that several nodes were different in controllability and synchronizability, without correction (Figure 4).

### 3.5 The correlation with clinical symptoms

We identified the regions with a significant correlation between average controllability, modal controllability, and synchronizability with the clinical symptoms in the frontal lobe, temporal lobe, and insular lobe. For the SZ patients (Table 1), the modal controllability and the BDRS scores exhibited significant negative correlations in the inferior frontal gyrus (*r* = −.375, *p* = .017) and a positive correlation in the fusiform gyrus (*r* = .335, *p* = .034). The SANS scores exhibited a significant positive correlation with modal controllability in the left insular gyrus (*r* = .383, *p* = .015) and a negative correlation with modal controllability posterior to the right superior temporal sulcus (*r* = −.414, *p* = .008) and left insular gyrus (*r* = −.449, *p* = .004). For BD patients (Table 2), the YMRS showed a significant positive correlation with modal controllability in the left thalamus (*r* = .349, *p* = .016), right inferior temporal gyrus (*r* = .323, *p* = .027) and left parahippocampal gyrus (*r* = .371, *p* = .010) and exhibited a significant negative correlation with synchronizability in the left inferior parietal lobule (*r* = −0.294, *p* = .045) and left parahippocampal gyrus (*r* = −.315, *p* = .031). The HAMD revealed a significant negative correlation with average controllability (*r* = −.318, *p* = .029) in the left thalamus and exhibited significant positive correlations with modal controllability in the left basal ganglia (*r* = .335, *p* = .021) and with synchronizability in the right basal ganglia (*r* = .302, *p* = .039).

**TABLE 1:** Spearman rank correlation coefficient (*p* value) of schizophrenia.

	Brain region	Correlation coefficient (*p* value)		
		BDRS	SANS	SAPS
Modal controllability	Inferior frontal gyrus _L_6_3	−0.375* (.017)	−0.055 (.736)	−0.148 (.363)
	Fusiform gyrus _R_3_2	0.335* (.034)	−0.055 (.737)	0.257 (.109)
	Posterior superior temporal sulcus _ R_2_2	0.015 (.927)	−0.414** (.008)	0.026 (.873)
	Insular gyrus _L _6_6	0.188 (.244)	0.383* (.015)	0.223 (.168)
Synchronizability	Parahippocampal gyrus _R _6_2	−0.146 (.370)	−0.419** (.008)	0.087 (.593)
	Insular gyrus _L _6_6	−0.130 (.422)	−0.449** (.004)	−0.177 (.275)

*p < .05, **p < .01

**TABLE 2:** Spearman rank correlation coefficient (p‐value) of bipolar disorder.

	Brain region	Correlation coefficient (*p* value)	
		YMRS	HAMD
Average controllability	Thalamus _L _8_4	−0.264 (.073)	−0.318* (.029)
Modal controllability	Basal Ganglia _L _6_6	0.180 (.225)	0.335* (.021)
	Thalamus _L _8_8	0.349* (.016)	0.216 (.145)
	Inferior Temporal Gyrus _R _7_3	0.323* (.027)	0.245 (.097)
	Parahippocampal Gyrus _L _6_4	0.371* (.010)	0.179 (.228)
	Basal Ganglia _R _6_2	0.008 (.956)	0.302* (.039)
Synchronizability	Inferior Parietal Lobule _R _6_5	−0.294* (.045)	−0.167 (.263)
	Parahippocampal Gyrus _R _6_2	−0.315* (.031)	0.017 (.910)

*p < .05

### 3.6 The classification

**FIGURE 5:**
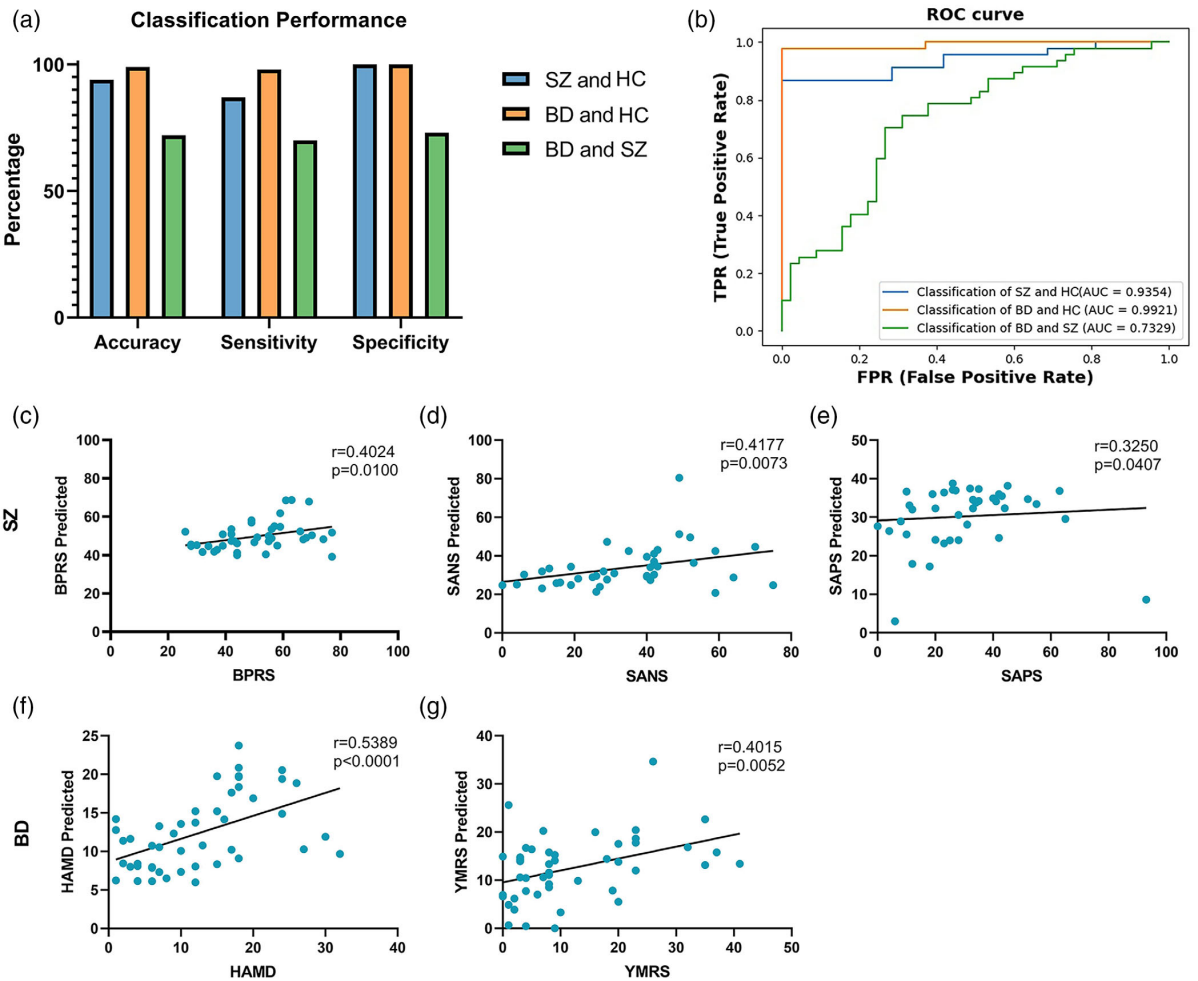
Classification performance and prediction of scale scores. (a) Accuracy, sensitivity and specificity. (b) Classification performance of classifiers with controllability and synchronization as factors. Scatter plots show correlations between the true scale scores and predictions made by a single linear model combining both positive and negative node sets of controllability and synchronizability. (c, d, e): The predicted score of the scale of SZ. (f, g): The predicted score of the scale of BD.

We observed the best classification performance in terms of the support vector machine between patients and the control group (Figure 5a,b). SZ and HC were classified with 94% accuracy and an area under the receiver operating characteristic curve (AUC) of 0.9354. BD and HC were classified with 99% accuracy and 0.9921 AUC. The above‐chance lower classification rate (accuracy: 72% and AUC: 0.7329) was found between the BD and SZ groups.

### 3.7 Prediction of the clinical scale

We found that controllability and synchronizability could be used to predict the scale scores in novel individuals with SZ (BDRS: *r* = .4024, *p* = .0100; SANS: *r* = .4177, *p* = .0073; SAPS: *r* = .3250, *p* = .0407) and BD (HAMD: *r* = .5389, *p* = .0001; YMRS: *r* = .4015, *p* = .0052), and the true and predicted scale scores were significantly correlated (Figure 5c–g).

## 4 DISCUSSION

Here, we showed abnormal white matter network dynamics in patients with SZ and BD, including impaired modal controllability and synchronizability but higher average controllability. The tradeoff decisions between controllability and synchronizability have changed. The supercontroller region and the control strategy of the cognitive system changed in patients with SZ and BD. We further found regional abnormities in the frontal, parietal, occipital lobe and subcortex, especially for modal controllability and synchronizability. The SZ and BD also showed differences mainly in controllability and synchronizability subcortex and parietal lobes. These abnormal controllability and synchronizability can classify patients and were significantly correlated with clinical symptoms.

### 4.1 Controllability and synchronizability of the white matter network in mental illness

We found that patients with SZ and BD showed abnormal white matter network dynamics, which indicated brain state changes (Cui et al., 2020). Our results implied that the network of patient groups required less energy to achieve easy‐to‐reach states and was easier to activate and required more energy to achieve difficult‐to‐reach states. There was an increase in average controllability in regions that involved auditory processing, language production and monitoring, and sensory information filtering, which might be associated with auditory verbal hallucinations in patients with SZ (Cui et al., 2017; Cui et al., 2020). The decrease in modal controllability implied an increase in driving energy for driving the brain to difficult‐to‐reach states in patients, which suggested that disruption to key control structures may represent a common biological substrate central to the pathophysiology of psychosis (Baker et al., 2014). The significant decreased synchronizability in the white matter network of patient groups means less ability to maintain a single state. Consistent with previous studies, SZ involves a disruption of neural synchronizability from the perspective of network properties (Zhu et al., 2020). Our results suggest that significant changes in controllability and synchronizability in patients with SZ and BD may be the cause of physiological dysfunction.

### 4.2 Relationship with controllability and synchronizability

We observed that modal controllability was positively correlated with average controllability and negatively correlated with synchronizability, which was consistent with previous studies (Tang et al., 2017). A person who is good at the state transition of one task is also good at the state transition of other tasks, while a person who is good at difficult‐to‐reach state transitions is not good at maintaining a single state (Tang et al., 2017). In patients with SZ and BD, the correction indices for controllability and synchronizability were altered. These correlations disappeared in the SZ group, indicating that the ability or flexibility to transition was decoupled in the SZ group. Although patients with BD showed a similar relationship with controllability and synchronizability, the correlation strength became weaker, which was quite different from the patterns of the HC group. Our results showed that the mutual patterns of controllability and synchronizability between the SZ group and BD group changed. In the present study, the findings implied that SZ and BD patients lost the optimal trajectory of state transfer (Gu et al., 2017) and changed the patterns in the ability of the brain to switch between different states.

### 4.3 Alteration of the supercontroller and control strategy

The patient groups showed additional supercontrollers of average controllability in the visual system, suggesting that the functional states are more easily activated in the occipital lobe (Muldoon et al., 2016). The regions could function to facilitate transitions to diverse states associated with these cognitive systems (Lee et al., 2019). In line with this, stronger activation of the occipital lobe has been proven to be associated with abnormal visual processing or positive symptoms in patients with SZ (Pirnia et al., 2015). The supercontroller with average controllability was preferentially located in the frontoparietal system and somatomotor system in the SZ group and BD group, respectively.

Patients with SZ and BD lose the supercontroller of modal controllability in the cingulate gyrus, especially patients with SZ. The cingulate gyrus is important for state transitions in the brain that require much cognitive work and higher executive functions (Cui et al., 2020), which are structurally and functionally deficient in the SZ and BD groups (Dufour et al., 2008; Fountoulakis et al., 2008; Knable et al., 2002). In addition, the thalamus became a supercontroller in patients, and modal supercontrollers that are mainly concentrated in the subcortical nuclei may be detrimental to higher‐order cognition (Tang et al., 2017).

Last, we observed more extensive synchronizability supercontrol areas in the patient groups, such as more extensive brain regions acting as controllers in the default system and fewer brain regions acting as controllers in the subcortical nuclei. Extensive synchronizability may inhibit the flexibility of different brain states (Tang et al., 2017), which is considered to be related to abnormal complex executive function (Medaglia et al., 2018). Cognitive systems play different control roles to achieve functional diversity (Anderson et al., 2013; Crossley et al., 2013). Our findings suggest that changing brain controllability strategies in a wide variety of ways, which may be the main cause of the abnormal function of mental illness.

### 4.4 Differences in regional metrics

The patients with both SZ and BD showed decreased modal controllability and increased synchronizability in the dorsal frontal lobe, temporal lobe, lobe parietal lobe, and occipital lobe. These brain regions associated with a lower ability drive their brains to difficult‐to‐reach functional states. Supporting our results, previous results showed a lower density of connectivity in the frontal, temporal and parietal cortex (Wheeler & Voineskos, 2014). A large number of studies have shown that abnormalities of the occipital lobe are related to SZ (Tohid et al., 2015), and the modal controllability reduction of the occipital lobe may affect the state transition ability beyond the occipital lobe functional states; Extensive synchronizability may cause abnormal control strategies (Tang et al., 2017), which is related to individual cognitive differences.

We also found that modal controllability increased and synchronizability reduced in the inferior parietal lobule, subcortical nuclei and insular lobe. The interruption of controllability and synchronizability indicated that the white matter network in parietal lobes was destroyed, which affected the local information communication among neurons (Li et al., 2019) and the optimal trajectory of brain state transitions (Gu et al., 2017). The relative strength of controllability between the subcortical and cortical regions is considered to be crucial for understanding individual differences in overall cognitive function (Tang et al., 2017), and the high modal controllability of the subcortical nucleus may be related to cognitive impairment. The insula plays an important role in sensory function and emotional processing (Pang et al., 2017), and the decrease in functional connectivity of the insula has been proven to be related to the severity of mental illness symptoms (Pang et al., 2017) and may also be the result of weakened structural connectivity and enhanced modal controllability.

We found that several nodes were different in controllability and synchronizability, between SZ and BD without correction. The average controllability of the right orbital gyrus and the synchronizability of the limbic system in SZ patients were lower than those in BD patients. In contrast, the modal controllability of the left temporal lobe and limbic system and the synchronizability of the inferior parietal lobule were higher than those in BD patients. Dysfunctions of the right orbital gyrus have been shown to be associated with emotional regulation (Zhao et al., 2020). Orbital gyrus and limbic system play a significant role in memory and emotion regulation (Rolls, 2015). Hyperactivation in inferior parietal lobule and temporal gyri might could imply as the potential state marker of schizophrenia (Soni et al., 2018). The abnormalities in the control ability of these brain regions we found may provide new directions for clinical diagnosis of disease types.

### 4.5 Relationships with the clinical symptoms

The severity of SZ was correlated with controllability and synchronizability in several regions. Modal controllability in the left inferior frontal gyrus showed negative correction with BDRS, which may affect the driving activities of the related brain in the cognitive process, resulting in mental disorder. There was a positive correlation between BDRS and the modal controllability of the right fusiform gyrus, which might be related to structural abnormalities and defects in the early stages of face perception in SZ (Onitsuka et al., 2003; Onitsuka et al., 2006). The SANS of patients with SZ positively correlated with modal controllability and negatively correlated with the synchronizability of the left insular gyrus. The insula plays an important role in sensory function and emotional processing (Pirnia et al., 2015), which may be associated with negative symptoms.

The severity of BD positively correlated with the modal controllability of the left thalamus, right inferior temporal gyrus and left parahippocampal gyrus and negatively correlated with the synchronizability of the right inferior parietal lobule and parahippocampal gyrus. The thalamus is the key structure for relaying and integrating information between the cortex and subcortical regions (Betzel et al., 2016), the increased modal controllability inhibited the ability of information exchange, which may be one of the reasons for the severity of BD. The severity of depressive symptoms of BD inversely correlated with the average controllability of the left thalamus and positively correlated with the modal controllability of the left basal ganglia, which might be involved in mood regulation (Lacerda et al., 2003). Abnormal average and modal controllability of the thalamus may affect the state activation of the neural pathway between the basal ganglia and thalamus, showing abnormal emotion regulation.

### 4.6 Classification and predictor performance

The average controllability, modal controllability and synchronizability could be used to classify patients from healthy controls with 94% accuracy, which higher than the recent neuroimage and brain network studies (Kambeitz et al., 2015; Lee et al., 2018; Schnack et al., 2014). Importantly, the SZ patients were also separated from BD patients with an accuracy of 72%, which meant that white matter network dynamic were different, especially for average controllability and synchronizability in the subcortex, parietal lobe and insular lobe. In addition, using improved CPM (Shen et al., 2017), we further demonstrated that controllability and synchronizability are powerful predictors of the clinical phenotype of mental illness. Our findings supported that controllability and synchronizability of the white matter network can serve as important metrics to judge the severity of mental illness.

## 5 LIMITATIONS

This study has potential limitations that should be considered when interpreting its conclusions. First, the sample size of this study was small, creating challenges for the interpretation and generalizability of results. Second, we use a time‐invariant, linear model of brain dynamics. However, the brain is highly non‐linear and explained well by models incorporating noise, although research has proved that the linear model can well simulate the dynamics of the brain(Gu et al., 2015). It remains an important and interesting direction to understand the control of brain dynamics based on nonlinear control methods.

## 6 CONCLUSION

We used network dynamics theory and control theory to analyze brain control strategies and found that the ability to drive brain state transitions was abnormal in patients with SZ and BD. The ability to drive the brain state to difficult‐to‐reach states was impaired, and it was easier to move to easy‐to‐reach states. The tradeoff decisions between controllability and synchronizability have changed. The brain regions that play a key role in driving the brain state shift and their distribution in the cognitive system were altered, and these changes in control strategies may be the reason for the difference in individual control ability. We were able to accurately discriminate patients with SZ and BD from HCs using abnormal controllability and synchronizability. This abnormal controllability or synchronizability was significantly associated with clinical symptoms and predicted the clinical symptoms. Our findings suggest that understanding the dynamic changes of white matter network is helpful to understand the physiological mechanism of mental illness and provide new guidance for the judgment and clinical intervention of mental illness.

## CONFLICT OF INTEREST

The authors declare no conflict of interest.

## Data Availability

The DTI data are available is available at OpenfMRI (https://openfmri.org/dataset/ds000030/). Code for metrics is available at: https://complexsystemsupenn.com/s/controllability_code‐smb8.zip.
